# *Toxoplasma gondii* alters NMDAR signaling and induces signs of Alzheimer’s disease in wild-type, C57BL/6 mice

**DOI:** 10.1186/s12974-018-1086-8

**Published:** 2018-02-23

**Authors:** Luisa Torres, Sudie-Ann Robinson, Do-Geun Kim, Angela Yan, Thomas A. Cleland, Margaret S. Bynoe

**Affiliations:** 1 Department of Microbiology and Immunology, College of Veterinary Medicine, Cornell University, Ithaca, NY 14853 USA; 2 Department of Psychology, Cornell University, Ithaca, NY 14853 USA

**Keywords:** Alzheimer’s disease, *Toxoplasma gondii*, NMDAR, Amyloid beta, Olfactory sensory neurons

## Abstract

**Background:**

Alzheimer’s disease (AD) is a progressive neurodegenerative disease associated with cognitive decline and complete loss of basic functions. The ubiquitous apicomplexan parasite *Toxoplasma gondii* (*T. gondii*) infects up to one third of the world’s population and is implicated in AD.

**Methods:**

We infected C57BL/6 wild-type male and female mice with 10 *T. gondii* ME49 cysts and assessed whether infection led to behavioral and anatomical effects using immunohistochemistry, immunofluorescence, Western blotting, cell culture assays, as well as an array of mouse behavior tests.

**Results:**

We show that *T. gondii* infection induced two major hallmarks of AD in the brains of C57BL/6 male and female mice: beta-amyloid (Aβ) immunoreactivity and hyperphosphorylated Tau. Infected mice showed significant neuronal death, loss of *N*-methyl-d-aspartate receptor (NMDAR) expression, and loss of olfactory sensory neurons. *T. gondii* infection also caused anxiety-like behavior, altered recognition of social novelty, altered spatial memory, and reduced olfactory sensitivity. This last finding was exclusive to male mice, as infected females showed intact olfactory sensitivity.

**Conclusions:**

These results demonstrate that *T. gondii* can induce advanced signs of AD in wild-type mice and that it may induce AD in some individuals with underlying health problems.

**Electronic supplementary material:**

The online version of this article (10.1186/s12974-018-1086-8) contains supplementary material, which is available to authorized users.

## Background

Alzheimer’s disease (AD) is a progressive neurodegenerative disease associated with decline in cognitive function [1, 2]. There are 5.1 million cases of AD in the USA and almost 2 million people suffering from dementia and cognitive decline [2]. This number will almost triple by the year 2050 due to increased numbers of individuals living beyond 85 years [2]. Major pathological hallmarks of AD include senile amyloid plaques composed of amyloid β (Aβ) protein and intracellular neurofibrillary tangles, with characteristic reactive microgliosis and astrogliosis [1, 2]. AD is also characterized by dystrophic neuritis, neuronal loss, synaptic dysfunction, and cerebral atrophy [1]. Viruses (herpes simplex virus 1), bacteria (*Chlamydia pneumoniae*), and parasites such as *Toxoplasma gondii* (*T. gondii*) are implicated in neurodegenerative diseases including Parkinson’s and AD [3, 4].

*T. gondii* is an obligate intracellular protozoan pathogen that enters the central nervous system (CNS) following its initial invasion and replication in the gut [5, 6]. *T. gondii* infects an estimated one third of the adult population worldwide, although seroprevalence varies significantly by country [7, 8] and is highly dependent on place of birth, educational level, living conditions, occupation, race, and ethnicity [9, 10]. Emerging studies implicate *T. gondii* as a contributor to Parkinson’s disease, schizophrenia, obsessive compulsive disorder, and Tourette syndrome [11]. Little is known about the long-term impact of *T. gondii* infection on neuronal cells, and their receptors such as the *N*-methyl-d-aspartate receptor (NMDAR), which is involved in synaptic plasticity and cognition. In response to neural activity, the NMDAR mediates the strength of synaptic transmission [12]. Synaptic loss is one of the major characteristics of AD and AD-related dementias [13]. Studies have shown that NMDARs mediate the downstream pathological effects of Aβ in animal models of AD. For instance, application of Aβ to neurons results in a rapid decrease in NMDAR function and expression [14]. This suggests that NMDAR is critical for maintaining neuronal function.

Glutamate, a major excitatory neurotransmitter, constitutes about 70% of all excitatory synapses in the CNS, and as such, it is tightly regulated [15]. Vesicular glutamate transporters, VGLUT1, 2, and 3, mediate the uptake of glutamate via synaptic vesicles [16]. VGLUT1 functions in neurotransmission while VGLUT2 plays a role in synaptic plasticity and protection from neuronal injury [17]. γ-Aminobutyric acid (GABA) is one of the major inhibitory neurotransmitters found in the adult brain and is synthesized from the conversion of glutamate via the catalytic decarboxylase GAD67. This mainly occurs in the presynaptic area of GABAergic neuron terminals in the brain [18]. Defects in GABAergic signaling are known to cause seizures and have links to AD, temporal lobe epilepsy, and schizophrenia.

We set out to determine whether *T. gondii* infection induces signs of AD in C57BL/6 (wild-type) mice including Aβ production, which is known to alter NMDAR signaling [19]. Here, we show that *T. gondii* causes signs of neurodegeneration in wild-type mice. We demonstrate that as infection progresses, *T. gondii* induces loss of NMDAR signal with concomitant induction of AD pathology including production of hyperphosphorylated Tau and Aβ immunoreactivity in the brain. We also observed neuronal death in the olfactory bulb accompanied by alterations in olfactory sensitivity which is known to occur in patients with AD [20]. The effects on the latter varied depending on sex: While olfactory sensitivity was reduced in male mice, females showed intact olfactory ability. Infected mice also showed alterations in social behavior, anxiety-like symptoms, and alterations in spatial learning. Thus, *T. gondii* can directly confer neurodegenerative and behavioral signs of AD in infected wild-type mice.

## Methods

### Mice

Eight- to 12-week-old C57BL/6 (wild-type) male and female mice were purchased from Jackson Laboratories. Mice were bred and housed under specific pathogen-free conditions in the animal facility at Cornell University.

### *T. gondii* infection in mice

Mice were randomly assigned to mock-infected and *T. gondii*-infected groups. *T. gondii* was orally infected following the infection protocol from Mahamed et al. [6]. Briefly, wild-type mice were infected with 10 *T. gondii* ME49 cysts which were maintained in Swiss Webster mice and isolated before infection. Survival rates and weight loss were monitored daily. For our experiments, groups of mice were euthanized at 15, 30, 60, and 90 days post infection and tissues (brain, spleen, spinal cord) were collected for further processing.

### Antibodies and reagents

Anti-APP antibody (6E10) was purchased from Covance (Cat# SIG-39320-1000). Anti-beta amyloid was purchased from Abcam (Cat#2539). Anti-*T. gondii* antibody was purchased from US biologicals (Cat# T8075-01). BAG1 was a gift from Dr. Dubey. Normal mouse IgG (SC-2025) and normal rabbit IgG (SC-2027) were used as isotype controls to test the specificity of the 6E10 and BAG1 antibodies, respectively. Anti-phospho-Tau antibody (AT8) was purchased from Thermofisher (Cat# MN1020). Anti-NeuN was purchased from Cell Signaling (Cat# 12943S) or Abcam (ab104224). Anti-VGLUT2 (Cat#71555S), anti-GAPDH (Cat# 2118), and anti-Tau (Cat#4019) antibodies were purchased from Cell Signaling. Anti-NMDAR (Cat# ab17345), anti-VGLUT1 (Cat# ab134283), and anti-GAD67 (Cat# ab26116) antibodies were purchased from Abcam. Thioflavin S was purchased from (Sigma Aldrich) and neuro-tracer from Life Technologies.

### Western blot

Immunoblots were performed on whole brain lysates from mock-infected and *T. gondii*-infected mice. The protein content was quantified by Bradford assay (Bio-Rad). Aliquots of samples were denatured and reduced with sodium dodecyl sulfate (SDS), and β-mercaptoethanol and equivalent concentrations of total protein were separated by SDS-PAGE. The proteins were transferred onto a nitrocellulose membrane. Non-specific protein binding was blocked by incubation with 5% bovine serum albumin (BSA) in phosphate-buffered saline (PBS) for 1 h at room temperature. The membranes were incubated with primary antibodies overnight at 4 °C (anti-NMDAR, anti-VGLUT1, anti-VGLUT2, anti-GAD67, anti-Tau, anti-phospho Tau, and anti-GAPDH), washed, and then probed with either goat anti-rabbit IgG human ads-HRP antibody (Southern Biotech, 1:2000 or goat anti-mouse IgG human ads-HRP antibody (Southern Biotech, 1:2000). Densitometry was performed on images of immunoblots for quantification with image J software.

### Preparation of samples for histology

Mice were terminally anesthetized using a ketamine-xylazine cocktail and perfused with ice-cold 4% paraformaldehyde (PFA), and tissues were collected and embedded with OCT. Samples were also flash frozen in liquid nitrogen for further analysis. For immunohistochemistry, sections were cut at 5–8-um thickness and post-fixed with 4% PFA.

### IHC and IFA

In general, frozen samples were washed twice with PBS and blocked with 10% goat serum/casein and incubated with anti-*T. gondii* primary antibodies, anti-RH strain or anti-BAG1 (1:1000), anti-NMDAR (1:200), anti-VGLUT1 (1:100), anti-VGLUT2 (1:50) or anti-GAD67 (1:100), anti-6E10 (1:200), anti-beta amyloid (1:200), anti-pTau (AT8) (1:1000), and anti-NeuN (1:200) overnight at 4 °C. Samples were washed twice with PBS and incubated with fluorochrome-conjugated secondary antibodies or horseradish peroxidase for 1 h for immunofluorescence assay (IFA) or 20 min for immunohistochemistry (IHC). For neurotracer staining, sections were incubated with neurotracer (1:500) for 30 min at room temperature. For IFA, samples were washed with PBS twice and coverslipped using vectashield with 4′,6-diamidino-2-phenylindole (DAPI) mounting media. For IHC, samples were washed with PBS twice then developed with AEC developing kit (Invitrogen) before counterstaining with hematoxylin. Slides were then coverslipped using fluoromount G mounting media. Images were captured using a Zeiss Axio Imager M1 microscope.

### Thioflavin S plaque staining

For thioflavin S plaque staining, frozen sections were fixed with 4% PFA, stained with primary antibody, anti-*T. gondii* antibody (1:1000). Samples were washed twice with PBS and incubated with fluochrome conjugated secondary antibody for 1 h. Samples were washed twice and incubated with 1% ThS (Sigma Aldrich) before differentiating in 70% ethanol. Slides were washed in distilled water mounted and coverslipped with DAPI. Sections were imaged and quantified for ThS^+^ immunoreactivity in infected and non-infected mice brains.

### Neuronal culture and *T. gondii* infection

Mouse embryonic cortical neurons were purchased from Life Technologies (Cat# A15586) and cultured for 6 days before infection as per manufacturer’s instructions. mCherry pru strain of *T. gondii* (kind gift of Dr. Denkers, Cornell University, Ithaca) was propagated in HS27 cells. Media of neuronal cells were changed every 3 days and cells were infected with 1 multiplicity of infection (MOI) of mCherry pru strain of *T. gondii* for up to 3 days. Cells were washed with ice-cold PBS twice and fixed with 4% PFA for 20 min and blocked with 5% goat serum for 1 h. Cells were then incubated overnight with anti-MAP-2 (MAB3418, Millipore, 1:200) and anti-NMDAR (Abcam, Cat# ab17345, 1:200) and washed twice with PBS. Cells were then incubated with fluorochrome conjugated secondary antibodies, washed, and coverslipped with prolonged gold DAPI for imaging.

### TUNEL assay

Terminal deoxynucleotidyl transferase dUTP nick end labeling (TUNEL) staining was used to detect apoptotic cells. The reaction mixture was supplied by Roche’s In Situ Cell Death Detection Kit, AP (Cat# 11684809910). Five- to eight-micrometer frozen sections were fixed with 4% paraformaldehyde for 20 min. Sections were then permeabilized using 0.1% Triton X-100 (Sigma-Aldrich, 9002-93-1) in sodium citrate (Fisher Scientific, 6132-04-3) with PBS for 2 min. Sections were blocked using 10% BSA dissolved in PBS. Primary antibody and TUNEL solution were added to each section. Antibodies included anti-NeuN (1:200) and anti-*Toxoplasma gondii* cysts (1:1000). Sections were left overnight at 4 °C. Positive and negative controls were prepared as instructed in the protocol. Sections were then incubated with secondary antibody (1:200) antibodies for 1 h. Following, sections were washed and coverslipped with vectashield with DAPI. Images were captured using a Zeiss Axio Imager M1 microscope.

### Open-field activity

Mobility in an open field was measured once during the animal’s light cycle at day 60 post *T. gondii* infection. Mock-infected mice were used as control. Each individual mouse was placed in the center of an open field arena (18 × 18 × 18 in.). The movement of the mouse was recorded by a USB webcam (Logitech HD-1820p) and a PC-based video capture software. The recorded video file was further analyzed by video tracking software (Topscan, Clever Systems) to determine the velocity and the total distance traveled by each mouse during the 5-min observation period. The percentage of time spent in the corners and in the center of the open field was also recorded. Both the test and the analysis were performed by an observer blinded to the treatment conditions.

### Sociability test

Sociability in mock-infected and *T gondii*-infected mice was measured once during the animal’s light cycle at day 60 post infection. We used a modified version of the Crawley’s sociability and preference for social novelty test that has been described previously [21]. The same open field arena (18 × 18 × 18 in.) used for the open field test was used to test social behavior. Two identical wired cups were placed in opposite sides of the arena. The test was divided into two 10-min sessions. In session 1, a mouse (stranger mouse 1) was placed under one of the wired cups, while the second wired cup located in the opposite side was left empty. The duration of the active contacts between the test mouse and both the empty cup and the cup containing stranger mouse 1 was recorded. An active contact was defined as any instance in which the mouse touched the wired cup with its snout or paws. In session 2, a second (novel) mouse was placed under the cup that had been empty during session 1. The duration of active contacts between the test mouse and both the familiar mouse and the novel mouse was recorded. The stranger mice and the subject mice were the same genotype, sex, weight, and age and were not littermates of each other. Both sessions were videotaped and manually analyzed by an observer blinded to the treatment conditions.

### Olfactory sensitivity test

Animals were habituated by placing each mouse in a clean, empty cage as described previously [22]. After 15 min, the mouse was moved to a new empty cage and allowed to habituate for 15 min. This process was repeated once more before the mouse was placed in the final cage for another 15 min prior to testing. The mouse was then presented with a square piece (2 × 2 in.) of filter paper that was impregnated with either an attractive scent (peanut butter, in concentrations ranging from 2.5 to 10%) or an aversive scent (10% trichloroacetic acid (TCA)). A piece of filter paper impregnated with water was used as control. The mouse was exposed to each scent for 3 min and the session was videotaped. The amount of time the mouse spent sniffing the filter paper was then recorded. The scents were presented in the following order: water, 2.5% peanut butter solution, 5% peanut butter solution, 10% peanut butter solution, and 10% TCA. After being exposed to a particular scent, each mouse was returned to a clean, empty cage for another round of habituation. Both the test and the analysis were carried out by an experimenter blinded to the treatment conditions.

### Barnes maze

Barnes maze was performed to assess spatial memory 60 days after infection with *T. gondii*. Mice were placed on the center of a wooden circular platform that was elevated 75 cm off the ground and that contained eight holes spaced 24.5 cm apart. An escape box measuring 10 × 8.5 × 4 cm was placed underneath a randomly selected hole. The platform measured 91 cm in diameter and the escape hole was 5 cm in diameter. The amount of time taken by the mouse to find the escape box (latency to find) and the amount of time taken to enter the escape box (latency to enter) were recorded. The session ended when the mouse entered the escape box or after 5 min. The mouse was left in the escape box for 1 min after entering and before being transferred back to the home cage. If the mouse did not find the escape hole within 5 min, it was placed into the escape box and left there for 1 min. Two trials separated by a 15-min inter-trial interval were performed daily for five consecutive days. The average latencies to find and enter the escape box were used for analysis.

### Experimental design and statistical analysis

All statistics were performed using GraphPad Prism 6 for Windows. All quantifications and analyses were performed by an observer blinded to the treatment conditions. Intensity quantification of NMDAR, Neurotracer, VGLUT1/2, and GAD67 were performed using Zen Digital Imaging software and were analyzed by one-way ANOVA followed by Bonferroni’s post hoc test. Quantification of 6E10^+^ signal, Thioflavine S^+^ signal, TUNEL^+^ neurons, TUNEL^+^ *T. gondii* cysts, and BAG1^+^ cysts was manually performed and analyzed using one-way ANOVA followed by Bonferroni’s post hoc test. Densitometry analysis of AT8, NMDA, VGLUT1/2, and GAD67 was performed using ImageJ and analyzed by one-way ANOVA followed by Bonferroni’s post hoc test. Open field data were analyzed by two-tailed Student’s *t* tests with Welch’s correction for samples having possibly unequal variances. Data from the social behavior test and the olfactory sensitivity test were analyzed by two-way ANOVA followed by Bonferroni’s post hoc test to correct for multiple comparisons. Barnes maze data were analyzed using two-way repeated measures ANOVA followed by Bonferroni’s post hoc test. The number of animals of each sex used in each experiment is specified in the figure legends. Data were presented as mean ± SEM. Data were considered statistically significant when *p* < 0.05.

## Results

### *T. gondii* associated with neurons and showed preference for infecting the olfactory bulb and the prefrontal cortex

**Fig. 1:**
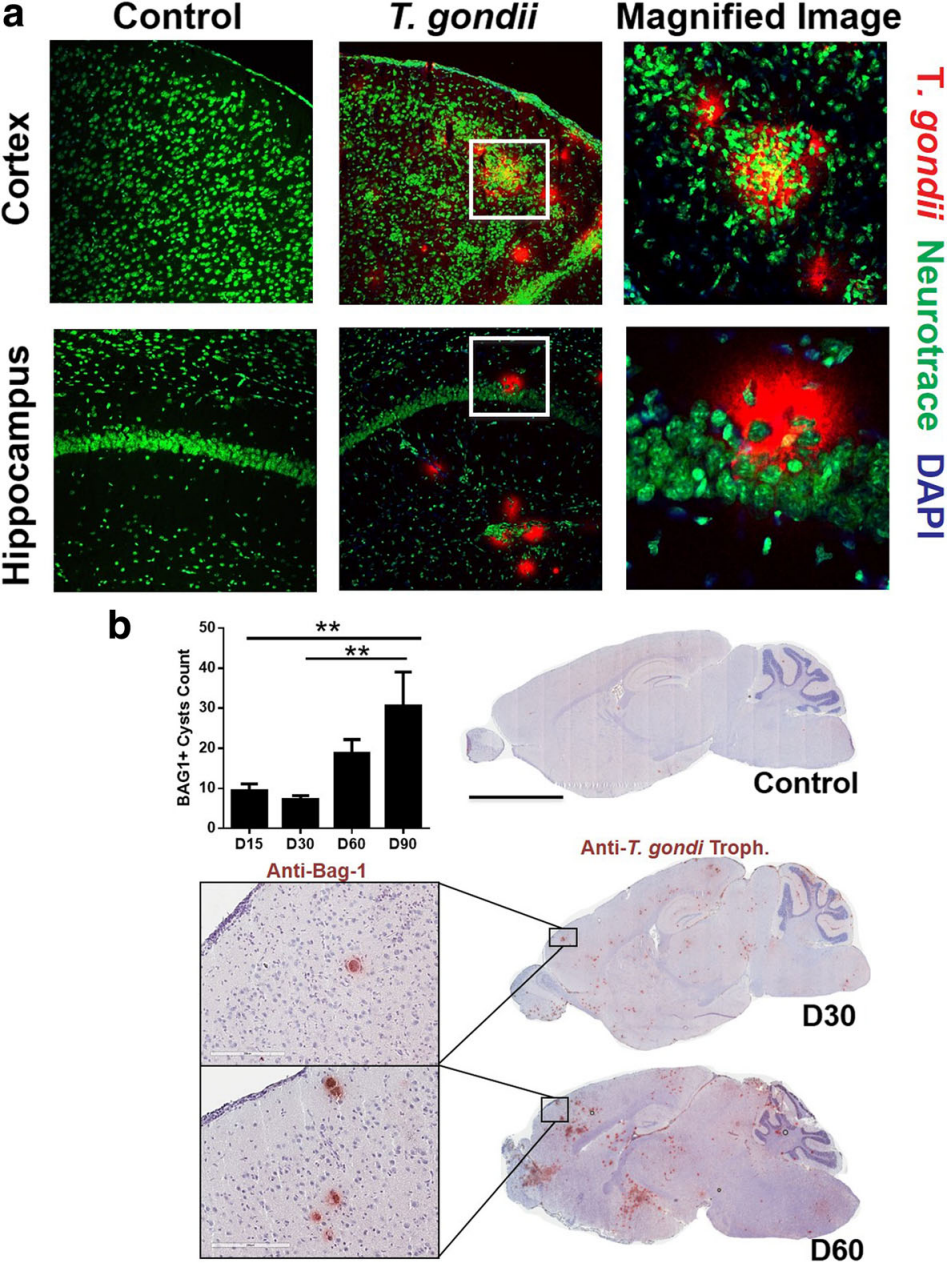
*T. gondii* associated with neurons in the brain. **a** Brains from mock-infected control mice or mice orally infected with 10 ME49 cysts after day 15 post infection were perfused with ice-cold PBS. Cryo-sections were fixed and stained with neurotracer (*green*) and anti-*T. gondii* antibody (*red*). Representative images from cortex and hippocampal areas of the brains of control and *T. gondii-*infected mice were captured and magnified images of boxed regions are shown on the right (*n* = 7). This experiment was repeated three times. **b** Whole brain scans of control and *T. gondii*-infected mice are shown for days 30 and 60 post infection. Zoomed in images show BAG1^+^ cysts in the cortical regions of the brain. Graph represents BAG1^+^ cyst counts for days 15, 30, 60, and 90 post infection (*n* = 3–4 mice per group). The experiment was repeated three times. Scale bar = 3 mm. Data are shown as mean ± SEM. *p < 0.05

The high prevalence of *T. gondii-*infected individuals and its association with neurodegenerative diseases led us to investigate whether the parasite had the capability to induce any of the major pathological signs of AD such as Aβ deposition or pTau in C57BL/6 wild-type mice. We infected wild-type mice with *T. gondii* and collected brains from control (mock-infected) and infected mice on days 15, 30, 60, and 90 post infection. Day 15 post infection is the pre-chronic infection stage when few parasites are found in the brain, while days 30, 60, and 90 represent the chronic (cyst) stage [23]. By day 15 post infection, we observed a few *T. gondii*-stained cysts associated with neurons (labeled with neurotracer) in the cortex and in the hippocampus of infected mice (Fig. 1a). There was a marked decrease in neurotracer intensity in the infected mice versus mock-infected controls. Some areas of the cortex showed no neurotracer signal, indicating a loss of neurons. Magnified images of the cortex and hippocampus showed an even more pronounced difference in neurotracer intensity. However, in control mice, those areas showed a more dense and compact neuronal staining (Fig. 1a). This suggests that *T. gondii* is associated with neuronal cells and may contribute to neuronal loss. Others have shown that *T. gondii* is not only associated with neurons [24, 25] but also with microglia and astrocytes [26–28]. To determine the distribution of *T. gondii cysts* throughout the brain, we scanned the entire brain of *T. gondii-*infected and mock-infected controls from days 15 to 90 post infection and quantified numbers of *T. gondii* cysts. Representative images of BAG1^+^ cysts in the cortex at days 30 and 60 are shown (Fig. 1b). We observed increasing frequency of *T. gondii* cysts stained with BAG1 antibody and/or *T. gondii* antigen at days 15, 30, 60, and 90 post infection (Fig. 1b). There were significantly more BAG1^+^cysts at day 90 compared to days 15 and 30 (*F*(3,12) = 8.487, *p* = 0.0027, one-way ANOVA). Moreover, *T. gondii* cysts were prominently clustered in specific brain regions (Fig. 1b), including the pre-frontal cortex and the olfactory bulb, two areas where others have observed cyst formation [29, 30]. These brain areas are also prominently associated with cognitive function and chemical signal response [31].

### *T. gondii* induced Aβ immunoreactivity in infected mice

**Fig. 2:**
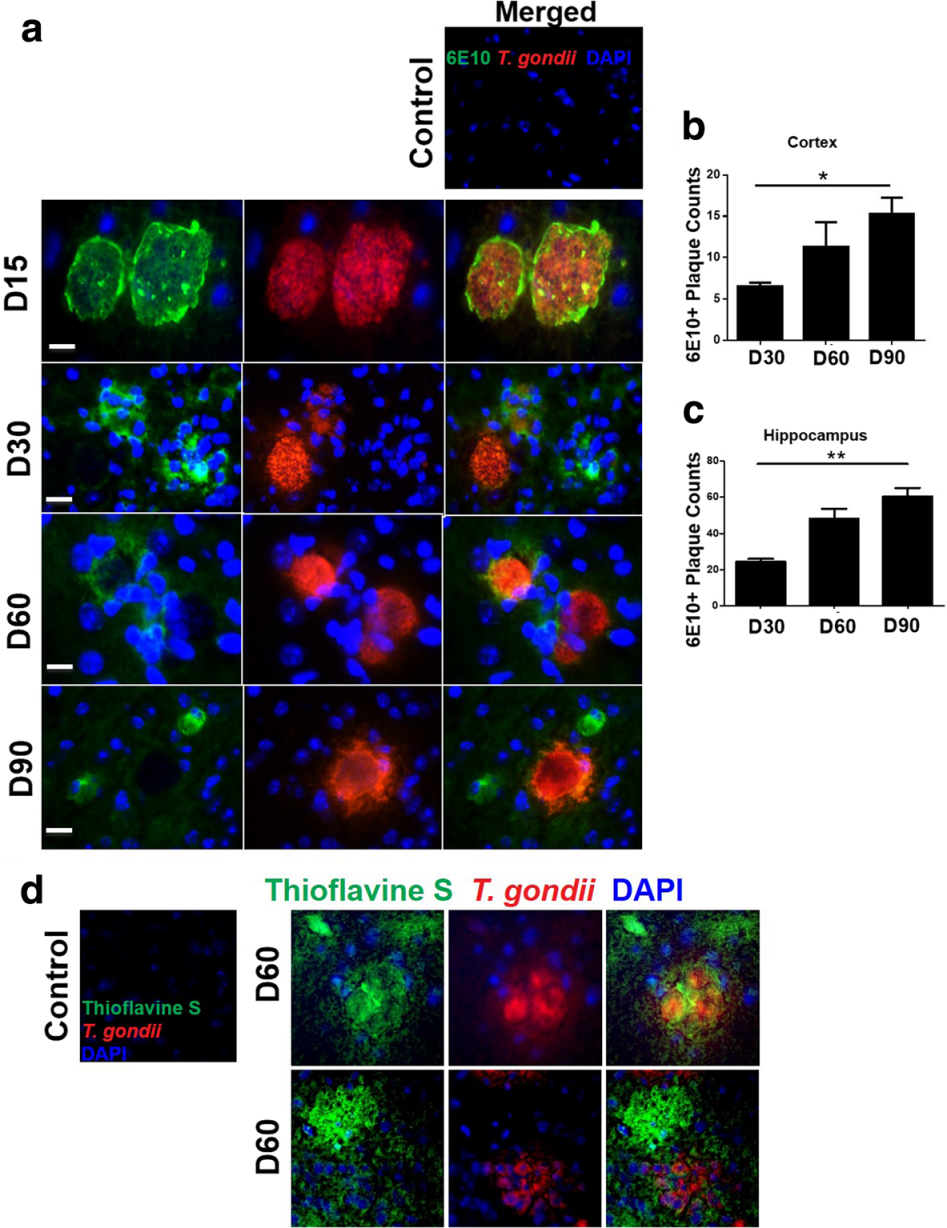
*T. gondii* infection induced Aβ immunoreactivity in the mouse brain. **a** Control brains and brains from mice orally infected with 10 ME49 cysts for 15, 30, 60, and 90 days were collected after perfusion with ice cold PBS. Sections were fixed and stained with a human anti-beta amyloid antibody, 6E10 (*green*) and an anti-*T. gondii* antibody BAG1 (*red*) and counterstained with DAPI (*blue*). *n* = 5 mice per group. Control sections were stained with anti-*T. gondii* antibody. **b** Quantification of 6E10-positive signal from 15 cortical regions from brains of mice at 30, 60, and 90 days post infection, *n* = 5 mice group. **c** Quantification of 6E10-positive signal from 10 hippocampal regions at 30, 60, and 90 days post infection. *n* = 5 mice per group. **d** Cryo-brain sections were stained with Thioflavin S (*green*) and anti-*T. gondii* (*red*) antibodies and counterstained with DAPI (*blue*). Representative images from *T. gondii* colocalized with 6E10 (*top*), as well as *T gondii* located distally from 6E10^+^ areas (*bottom*) are shown; *n* = 2–3 mice per group. Data are shown as mean ± SEM. *p < 0.05, **p < 0.01

We next determined whether *T. gondii* infection induced signs of AD in wild-type mice. We therefore analyzed the brains of *T. gondii-*infected mice for signs of Aβ plaques, which is one of the major hallmarks of AD (Fig. 2). We stained brains from *T. gondii-*infected mice and mock-infected controls with 6E10, an antibody that is reactive to amino acid residue 1–16 of human beta amyloid [32]. Intriguingly, we observed that the brains of *T. gondii-*infected mice stained positive for 6E10, whereas no sign of 6E10 staining was observed in mock-infected controls (Fig. 2a and Additional file 1: Figure S1). Moreover, there was co-localization of 6E10 and BAG1-positive *T. gondii* cysts as early as day 15 post infection (Fig. 2a). We next determined whether Aβ staining co-localized only with *T. gondii* cysts or whether widespread Aβ immunoreactivity was found in other areas of the brain where *T. gondii* cysts were not present. Indeed, we observed Aβ immunoreactivity in other areas of the brain where they did not co-localize with cysts at days 60 and 90 post infection (Fig. 2a, d). One-way ANOVA did not reveal a significant difference in 6E10^+^ signal (*F*(2,6) = 3.064. *p* = 0.1211). However, direct group comparisons between 6E10^+^ signal at days 30 and 90 showed that 6E10^+^ signal in the cortex increased significantly (*p* = 0.0450, two-tailed student’s *t* test, Fig. 2b). 6E10^+^ signal also increased significantly in the hippocampus from day 30 to day 90 (*F*(2,7) = 10.22, *p* = 0.0084, one-way ANOVA. Fig. 2c). By 90 days post infection, quintessential plaque morphology was seen with 6E10 staining (Fig. 2a) surrounding cysts and also near cysts. We stained day 60 brain sections with normal mouse IgG and normal rabbit IgG to confirm antibody specificity of the 6E10 and BAG1 antibodies, respectively. We observed no 6E10 or BAG-1 signal in the brains at day 60 post infection, indicating that the antibodies were specific (Additional file 1: Figure S1). To further confirm the presence of Aβ immunoreactivity, we next stained brains of *T. gondii-*infected and mock-infected mice with Thioflavin S, a dye that fluoresces an apple green color when specifically bound to Aβ (Fig. 2d). We stained day 60 infected mice brains with Thioflavin S and observed strong Thioflavin S immunoreactivity surrounding *T. gondii* cysts, confirming that *T. gondii* induces Aβ immunoreactivity in infected mice but not in mock-infected controls (Fig. 2d).

Since the 6E10 antibody we used is reactive to amino acid residues 1–16 of human beta amyloid, we wanted to test whether we could see Aβ plaques in our mouse samples when using an antibody reactive to mouse beta amyloid (anti-beta amyloid antibody, Abcam (Cat#2539)). We observed anti-beta amyloid signal in the brain cortex at days 30 and 60 post infection and found BAG-1 signal in comparable regions of the cortex. (Additional file 2: Figure S2A). We also observed 6E10 signal in tissue sections from the same animals we used to look for mouse Aβ but did not find any 6E10 signal when using isotype controls (Additional file 2: Figure S2B). Together, these findings show that *T. gondii* infection was capable of inducing signs of neurodegeneration, as well as, Aβ immunoreactivity, which is one of the major hallmarks of AD.

### *T. gondii* infection induced Tau hyperphosphorylation

**Fig. 3:**
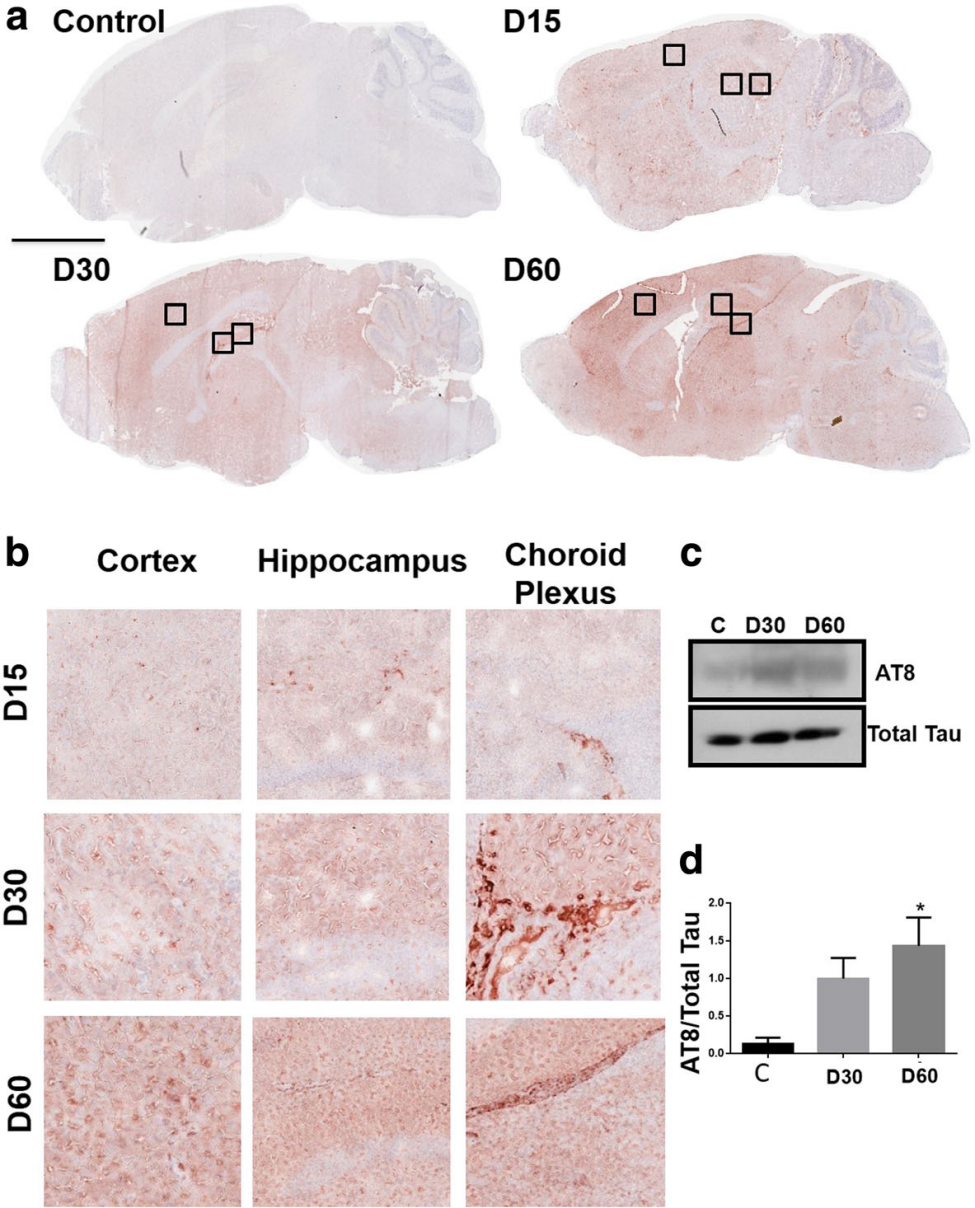
*T. gondii* infection induced Tau phosphorylation in the mouse brain. **a** Brains from mock-infected control mice and mice orally infected with 10 ME49 cysts for 15, 30, and 60 days were collected after perfusion with ice cold PBS. Brain sections were fixed and stained with an anti-AT8 antibody that specifically recognizes phosphorylated Tau (pTau) and was visualized after the reaction with an AEC substrate-developing kit (Invitrogen). *n* = 2–3 mice per group. Scale bar = 3 mm. **b** Magnified images of squared regions from cortex, hippocampus, and choroid plexus are shown for each time point. *n* = 5 mice per group. **c** Western blot analysis of AT8 (79 kDa) protein levels using whole brain samples from mock-infected controls and *T. gondii*-infected mice at days 30 and 60 post infection. Blot shown is representative of three independent experiments. **d** Densitometric analysis of Western blot data from **c**. The results are expressed as the ratio of densities of AT8-specific bands to total Tau bands (50–80 kDa). Data are shown as mean ± SEM. **p < 0.01

We investigated whether *T. gondii* infection induced Tau hyperphosphorylation (pTau), another major hallmark of AD [33]. Tau is a microtubule-associated protein that is involved in the transport of neurotransmitters through axons [33]. Phosphorylation of Tau protein forms neurofibrillary tangles in neuronal cells causing collapse of the cytoskeleton, thereby hindering neurotransmission. To determine whether *T. gondii* induced pTau expression, we performed immunohistochemistry using the AT8 antibody which specifically stains pTau [34]. We observed pTau staining in the brains of *T. gondii-*infected mice beginning at day 15, with increased staining intensity by day 60 post infection (Fig. 3a, b). The intensity of pTau signal increased as the infection progressed, thus, day 60 infected mice had significantly stronger pTau staining compared to the brains at day 15 or 30 post infection (Fig. 3a, b). This was most pronounced in the cortex and hippocampus, which are areas that are critically involved in memory and cognition [35, 36]. We also saw strong pTau staining in the choroid plexus of infected animals at day 30 (Fig. 3b). We confirmed these results by Western blot using protein samples from the brains of mock-infected and *T. gondii*-infected mice at days 30 and 60. We found a significant increase in protein levels of AT8 in the brains of infected mice at day 60 post infection (*F*(2,9) = 5.895, *p* = 0.0231, one-way ANOVA. Fig. 3c–d). Based on these cumulative data, we conclude that infection by *T. gondii* directly induces major pathological hallmarks of AD.

### *T. gondii* infection led to loss of NMDAR signal

**Fig. 4:**
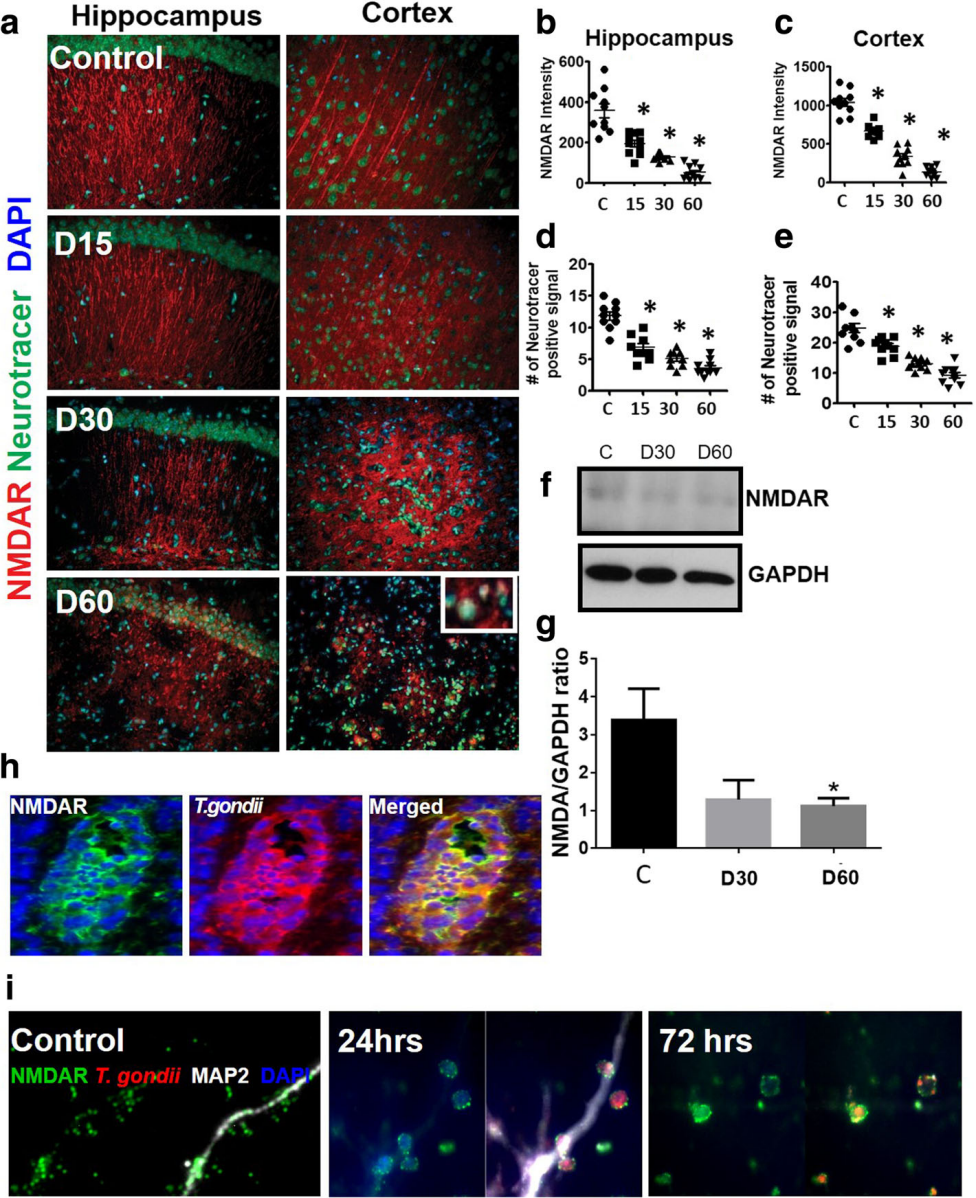
*T. gondii* infection disrupted expression of the NMDA receptor in the mouse brain. **a** Brains from mock-infected control mice and mice orally infected with 10 ME49 cysts for 15, 30, and 60 days were collected after perfusion with ice-cold 4% PFA. Brain sections were fixed and stained with neurotracer (*green*), anti-NMDAR antibody (*red*), and counterstained with DAPI (*blue*). Representative images were taken from hippocampal and cortical regions at × 20 magnification *n* = 3–4 mice/group. The experiment was repeated twice. The inset image from day 60 shows co-localization of NMDAR and neurotracer. **b**, **c** Intensity of NMDAR (*Texas Red*) from hippocampus (**b**) and cortex (**c**) at different times post infection were analyzed. Five different fields were randomly selected from two different animals per group. **d**, **e** Number of neurotracer positive cells (AF488) from hippocampus and cortex, respectively. *n* = 2–4 per group. **f** Western blot analysis of NMDAR (105 kDa) protein levels using whole brain samples from mock-infected controls and *T. gondii*-infected mice at days 30 and 60 post infection. GAPDH (37 kDa) was used as loading control. Blot shown is representative of three independent experiments. **g** Densitometric analysis of Western blot data from **f** using ImageJ. The results are expressed as the ratio of densities of NMDAR-specific bands to GAPDH bands. **h** Brain sections from infected mice were stained with antibodies to *T. gondii* (*red*) and NMDAR (*green*). Brain sections were counterstained with DAPI (*blue*). **i** Mouse cortical neuronal cells (E15 cortical neurons) were infected with 1 MOI of *T. gondii* (mCherry pru strain, *red*) for 24–72 h. Infected neurons were fixed, permeabilized, and stained with anti-MAP-2 (*white*) and anti-NMDAR **(***green*) antibodies. Cells were counterstained with DAPI (*blue*). Representative images of the axons of infected neurons 24–72 h post infection are shown. Images are representative of two independent experiments. Data are shown as mean ± SEM. *p < 0.05

Glutamate is the major excitatory neurotransmitter in the CNS and it is released at a high rate at synapses in the brain. Glutamate may be endocytosed or released by glutamatergic NMDARs that are widely expressed on neuronal cells and functions in maintaining CNS homeostasis [37, 38]. NMDAR plays a predominant role in controlling synaptic plasticity including learning and memory function; and NMDAR dysfunction is widely implicated in the pathophysiology of AD [39]. Given the strong evidence showing that Aβ causes neuronal pathology and dysfunction that can be abrogated in the presence of NMDAR antagonism [40], we hypothesized that the Aβ immunoreactivity found in infected mice may alter NMDAR expression. We examined the brains of infected and mock-infected wild-type mice on days 15, 30, and 60 post infection to evaluate NMDAR expression with an antibody against the NMDAR subunit 1 (NR1) and co-stained with neurotracer. Intriguingly, at days 15 and 30, we observed a significant decrease in NMDAR expression intensity especially in the cortex and hippocampus, when compared to controls (Fig. 4a–c). This effect was even more pronounced at day 60, which showed a dramatic decrease in NMDAR expression (Fig. 4a–c). Loss of NMDAR signal was also consistent with a significant decrease in neuronal signal suggesting that NMDAR loss was related to neuronal loss (Fig. 4a, d, e). We also observed that NMDARs co-localized directly with *T. gondii* cysts, further supporting the notion that *T. gondii* is responsible for NMDAR loss (Fig. 4h). We tested the effect of *T. gondii* infection on protein levels of NMDAR by Western blot using protein samples from the brains of mock-infected and *T. gondii*-infected mice. We found significantly reduced protein levels of NMDAR in the brains of infected mice at day 60 post infection (*F*(2,12) = 4.906, *p* = 0.0277, one-way ANOVA, Fig. 4f, g). We also observed that NMDARs co-localized directly with *T. gondii* cysts, further supporting the notion that *T. gondii* is responsible for NMDAR loss (Fig. 4h). To further confirm whether *T. gondii* infection directly caused loss of NMDAR on neurons, we infected neuronal cells in vitro with 1 MOI of *T. gondii* (mCherry pru strain). Mock-infected cells were used as controls. We observed a similar decrease in NMDAR expression on infected neurons for up to 72 h post infection (Fig. 4i). Intriguingly, any remaining NMDAR expression was tightly clustered around *T. gondii* cysts both in vivo and in vitro, and by 72 h, there was almost complete loss of NMDAR signal (Fig. 4i). This decrease in NMDAR signal may be similar to NMDAR antagonism that may lead to receptor under-excitation and hypofunction resulting in cognitive impairment, similar to what is observed in AD [15]. Since *T. gondii* cysts were in close contact with NMDAR (Fig. 4i), it is reasonable to hypothesize that the parasite interaction with NMDAR hindered normal neurotransmission, although this hypothesis needs further investigation. The potent downregulation or loss of NMDAR is strongly indicative of the Aβ-induced synaptic depression and impaired synaptic plasticity that is seen in AD [41, 42].

### *T. gondii* infection led to loss of VGLUT2, which is indicative of loss of olfactory sensory neurons

**Fig. 5:**
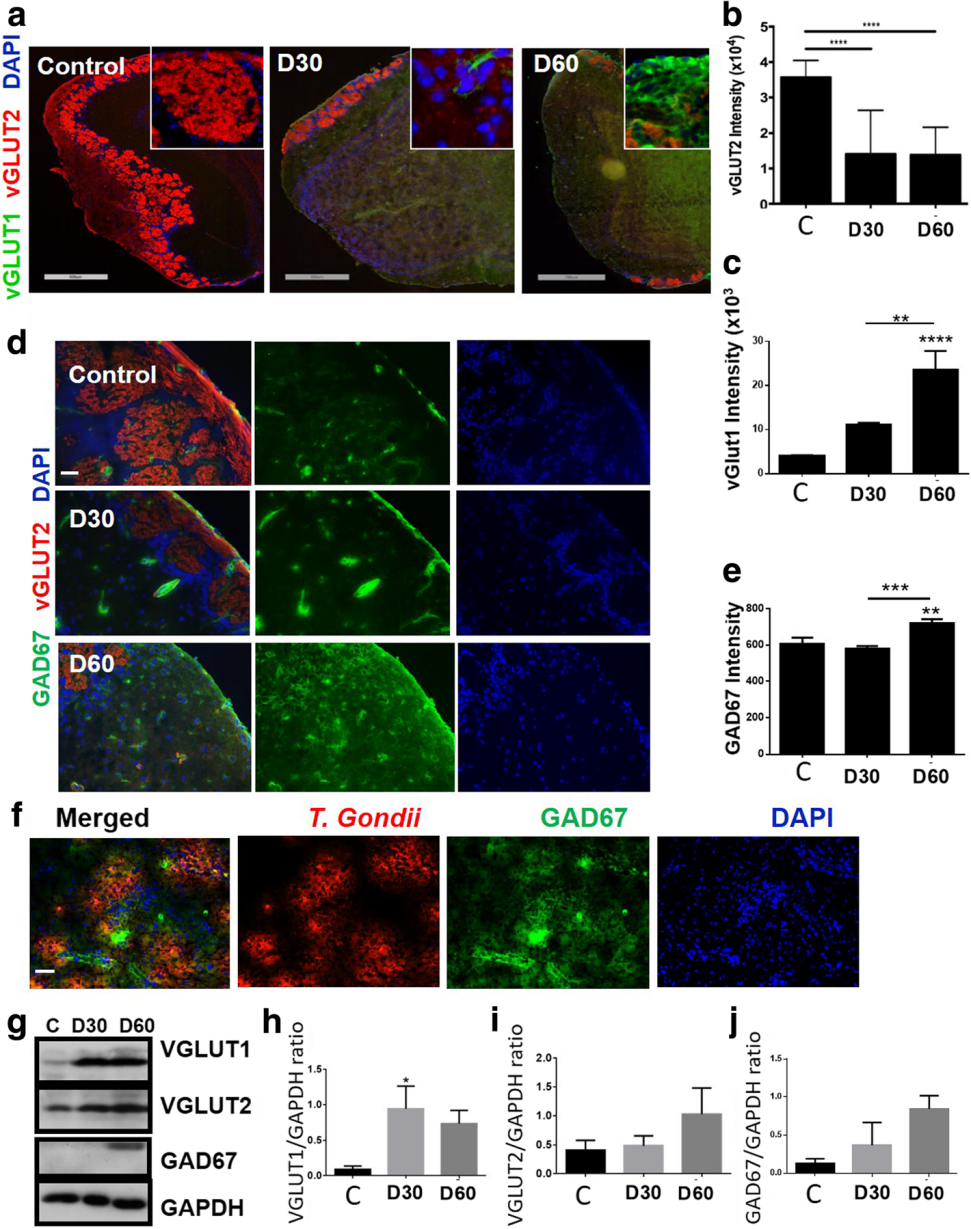
*T. gondii* infection decreased VGLUT2 expression in the mouse olfactory bulb. **a** Brains from mock-infected control mice and mice orally infected with 10 ME49 cysts for 30 and 60 days were collected after perfusion with ice-cold 4% PFA. Brain sections were fixed and stained with anti-VGLUT1 (*green*), anti-VGLUT2 (*red*), and mounted with DAPI (*blue*). Whole brain images were scanned using Leica Biosystems Scanscope and processed with image scope. Representative images were taken from the olfactory bulb at × 20 magnification. *n* = 4–5 mice per group, the experiment was repeated three times. Scale bar = 50 μm. **b**, **c** At least five different fields of the olfactory bulb and pre-frontal cortex were randomly selected from 2 to 4 mice per group. The intensity of VGLUT2 (**b**) and VGLUT1 (**c**) signal at different time points post infection was quantified. **d** Representative images of the olfactory bulb of mock-infected and *T. gondii-*infected mice stained with anti-GAD67 (*green*) and VGLUT2 (*red*). Scale bar = 50 μm, *n* = 4 mice per group. **e** The mean fluorescent intensity for GAD67 (*green*) was measured using Axiovision software from five different fields (*n* = 4 mice per group). **f** Representative image from the olfactory bulb of infected mice stained with anti-GAD67 (*green*) and anti-*T. gondii* (*red*). Scale bar = 50 μm. **g** Western blot analysis of VGLUT1/2 (55 and 65 kDa, respectively) and GAD67 (67 kDa) protein levels using whole brain samples from mock-infected controls and *T. gondii*-infected mice at days 30 and 60 post infection. GAPDH (37 kDa) was used as loading control. Blot shown is representative of three independent experiments. **h–j** Densitometric analysis of Western blot data from **g**. The results are expressed as the ratio of densities of GAD67 or VGLUT1/2-specific bands to GAPDH bands. Data are shown as mean ± SEM. *p < 0.05, **p < 0.01, ***p < 0.001, **** *p* < 0.0001

Glutamate uptake and transport are mediated by vesicular glutamate transporters (VGLUTs). VGLUT1 functions in neurotransmission and VGLUT2 functions in synaptic plasticity. In AD patients, VGLUT expression and function are impaired in the pre-frontal cortex [43], and olfactory dysfunction and atrophy are one of the early signs of AD [20]. Olfactory dysfunction causes a loss of sensory input in the entorhinal cortex and hippocampus [20]. The excitatory VGLUT2 is a molecular marker for glutamatergic olfactory sensory neurons, which transmit information from the nose to the brain [44, 45]. Since the olfactory bulb was heavily infected with *T. gondii* cysts, and olfactory dysfunction is one of the early indicators of AD, we examined VGLUT2 expression in the olfactory bulb on days 30 and 60 post infection. Compared to control mice that expressed their full complement of VGLUT2 in the olfactory glomeruli, we observed that more than two thirds of olfactory VGLUT2 expression were lost in *T. gondii-*infected mice on days 30 and 60 (Fig. 5a, b). This loss of olfactory VGLUT2 is indicative of extensive loss of olfactory sensory neurons in infected mice. Interestingly, there was also a notable decrease in VGLUT2 expression in the hippocampus where *T. gondii* cysts were also present (data not shown). This dramatic loss of VGLUT2 expression in the olfactory glomeruli is indicative of severe glutamatergic dysfunction and disturbance of synaptic plasticity that usually leads to cognitive impairment. On the other hand, VGLUT1 expression significantly increased in areas of the brain where VGLUT2 expression was decreased, suggesting that VGLUT1 may be compensating for the loss of VGLUT2 in these areas (*F*(2,27) = 15.78, *p* < 0.0001, one-way ANOVA, Fig. 5a, c). Western blots using whole-brain samples from control and infected mice at day 60 revealed unchanged levels of VGLUT2 protein (*F*(2,14) = 1.422, *p* = 0.2739, one-way ANOVA, Fig. 5g, i) and significantly increased levels of VGLUT1 in the infected brains compared to controls (*F*(2,14) = 4.327, *p* = 0.0344, one-way ANOVA, Fig. 5g, h).

### *T. gondii*-infected mice showed a discrete spatial increase in GAD67 expression

GAD67 (l-glutamic acid decarboxylase67) is one of two enzymes that generates γ-amino butyric acid (GABA). GAD67 is responsible for synthesizing more than 90% of GABA in the brain and its activity is rate limiting [46]. Alteration in GABAergic neurotransmission is associated with AD, as an increase in the expression of GAD67 was found in the brains of AD patients and in AD transgenic mice [47]. To determine whether GABAergic signaling was altered in infected mice, we examined the expression level of GAD67 in the brains of mock-infected and *T. gondii-*infected mice on days 30 and 60 post infection. GAD67 was upregulated in discrete regions of the infected mouse brain including the olfactory bulb (Fig. 5d–e). The overall expression of GAD67 was significantly increased over control at day 60 post infection (*F*(2,24) = 10.45, *p* = 0.0005, one-way ANOVA). Protein levels of GAD67 were also moderately increased at days 30 and 60 post infection (*F*(2,3) = 2.002, *p* = 0.2803, one-way ANOVA, Fig. 4g, j). In a recent report, GAD67 expression was displaced from the synaptic termini and redistributed throughout the neuropil in *T. gondii*-infected mice [48]. Thus, it is plausible that GAD67 increase is an attempt to re-establish GABAergic nerve terminals in areas where neurotransmission is most deficient. Interestingly, the increased GAD67 expression occurred at sites where *T. gondii* cysts were most prevalent (Fig. 5f), suggesting that *T. gondii* cyst formation caused alterations in intracellular GABA production. Furthermore, this is consistent with previous reports that showed that *T. gondii* uses GABA as its carbon source for its metabolism [49].

### *T. gondii* cysts were associated with neuronal cell death

**Fig. 6:**
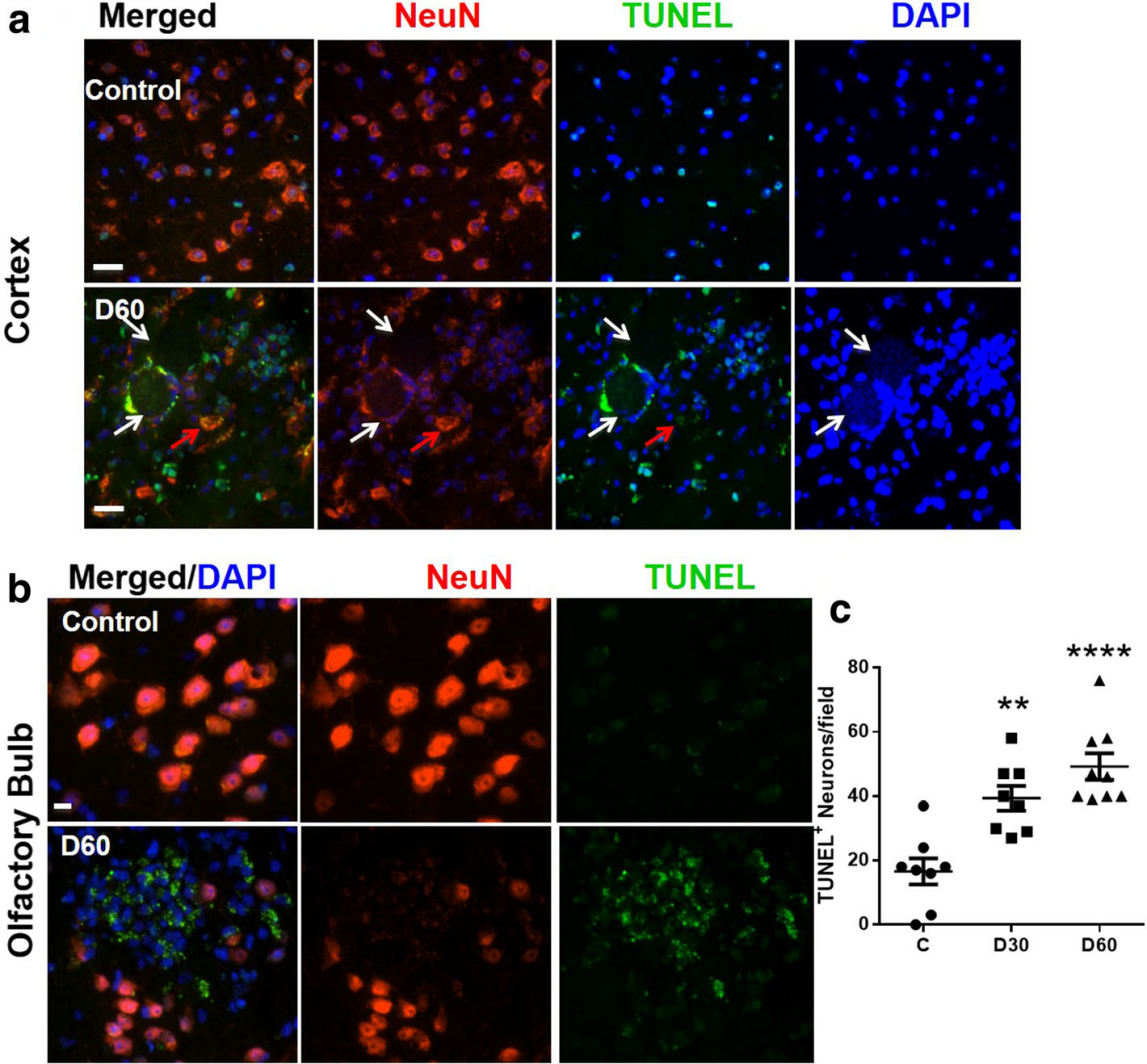
*T. gondii* infection caused neurodegeneration in the mouse olfactory bulb and the pre-frontal cortex. Brains from mock-infected (control) mice and mice orally infected with 10 ME49 cysts for 30 and 60 days were collected and prepared as previously noted. **a** Brain sections (*n* = 4 mice per group) were fixed and stained with TUNEL (*green*), anti-NeuN (*red*), and DAPI (*blue*). Representative images from biological replicates were taken from the prefrontal cortex at ×20 magnification. *n* = 4 per group, scale bar = 20 μm. White arrows point to areas where *T. gondii* cysts are present, while red arrows show a NeuN^+^ TUNEL^+^ neuron in the vicinity of the cyst. **b** Representative cortical areas devoid of cysts that show significant presence of NeuN^+^ TUNEL^+^ neurons. *n* = 4 mice per group. **c** At least five different fields from the olfactory bulb and pre-frontal cortex were randomly selected from 2 to 3 mice/group and the number of TUNEL^+^ NeuN^+^ cells was quantified

To determine whether *T. gondii* infection led to cell death in infected mice, we pinpointed areas in the prefrontal cortex that specifically contained *T. gondii* cysts and double stained with antibodies to both TUNEL and NeuN. We observed co-localization of both TUNEL and NeuN in the areas where *T. gondii* cysts were present (Fig. 6a). This indicates that *T. gondii* induced neuronal cell death. In addition, the number of NeuN^+^ TUNEL^+^ cells increased significantly as infection progressed (*F*(2,22) = 17.20, *p* < 0.0001, one-way ANOVA, Fig. 6c). We also observed areas in the olfactory bulb that were positive for TUNEL but not for NeuN, suggesting that other cell types were also undergoing apoptosis (Fig. 6b). However, the percentage of NeuN^+^ TUNEL^+^ cells was significant after infection compared to the control. We surmise these other cell types are astrocytes and/or microglia as previous reports have shown significant damage to these cells post infection [36]. We next asked whether similar cell death occurred in the olfactory bulb, which was heavily infected with *T. gondii* cysts. Indeed, we observed strong TUNEL^+^ staining in the entire olfactory bulb (Fig. 6b), suggesting that neuronal cells in the olfactory bulb were undergoing cell death, which is consistent with the loss of olfactory VGLUT2 staining in the olfactory glomeruli (Fig. 5a). Moreover, we observed that some areas of the olfactory bulb were devoid of cells noted by the loss of DAPI staining (data not shown), indicating that cell death had already occurred in those areas. Control sections show the presence of cells in these olfactory regions.

### *T. gondii*-infected mice had altered social memory and displayed anxiety-like behavior 60 days after infection

**Fig. 7:**
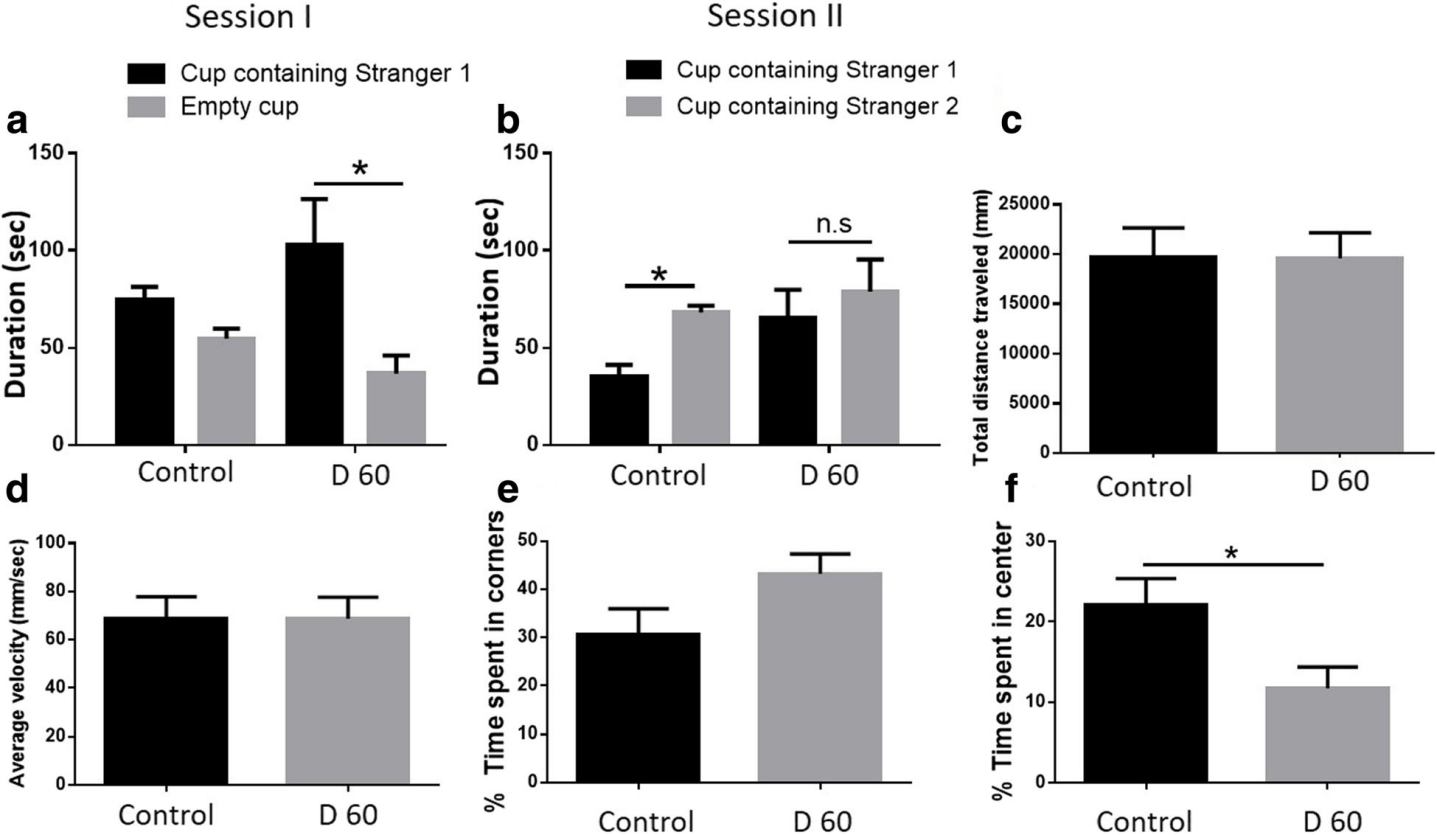
*T. gondii* infection altered social novelty recognition and caused anxiety-like symptoms. **a** Mice were tested once on day 60 after infection with *T. gondii*. A modified version of the Crawley’s sociability test was used. The duration of active contacts between the test mouse and the empty cup or the cup containing stranger mouse 1 is shown. *n* = 9–13 per group (control group: 5 males and 4 females; experimental group: 6 males and 7 females). **b** Duration of active contacts between the test mouse and stranger mouse 1 (familiar mouse) and stranger mouse 2 (novel mouse), *n* = 9–13 per group (control group: 5 males and 4 females; experimental group: 6 males and 7 females). **c** The total distance traveled, (**d)** the average velocity, and (**e)** the percentage of time spent in the corners and in the center (**f**) of the open field during the 5-min observation period were recorded. Mice were tested once at day 60 post *T. gondii* infection on the same day social behavior was measured. *n* = 6–10 per group (control group: 2 males and 4 females; experimental group: 3 males and 7 females). Data are shown as mean ± SEM. *p < 0.05

With disruption of NMDAR, alterations of GAD67 and extensive damage to neuronal cells, we set out to determine if there were any behavioral changes affecting mice infected with *T. gondii* cysts. We observed *T. gondii* clusters in both the prefrontal cortex and the hippocampus, two brain regions that have been linked to regulation of social behavior [50, 51]. To determine whether the effects of *T. gondii* infection extended to changes in social behavior, we used a modified version of the Crawley’s sociability and preference for social novelty test previously described [21]. Mock-infected mice displayed a preference for the cup containing stranger mouse 1 over the empty cup, which is indicative of normal social behavior (Fig. 7a). Two-way ANOVA revealed that *T. gondii*-infected mice spent significantly more time with the cup containing stranger mouse 1 over the empty container, also indicative of normal behavior (*F*(1,40) = 7.553, *p* = 0.0089, Fig. 7a). Specific two-group comparisons revealed that control mice preferred the container with the novel mouse (stranger mouse 2) over the familiar mouse (stranger mouse 1) in the second phase of the experiment, and this difference was significant (*p* = 0.0229, two-tailed Student’s *t* test). However, *T. gondii*-infected animals did not show a preference for either container, indicating an alteration in recognition of social novelty (*p* = 0.5461, two-tailed Student’s *t* test, Fig. 7b).

To understand whether the observed effect was mediated by hyperactivity or motor impairments in the infected animals, we assessed the activity of the same cohort of animals in an open field test environment. *T. gondii* infection did not affect the distance traveled (Fig. 7c, *p* = 0.9669, two-tailed Student’s *t* test) or average velocity (Fig. 7d, *p* = 0.9866, two-tailed Student’s *t* test) in the open field. However, *T. gondii*-infected animals spent more time in the corners of the open field (Fig. 7e, *p* = 0.0896, two-tailed Student’s *t* test) and significantly less time in the center compared to mock-infected controls (Fig. 7f, *p* = 0.0365, two-tailed Student’s *t* test). This behavioral outcome has been associated with increased anxiety [52] and is also common among AD patients [53]. Taken together, these results indicate that *T. gondii*-infected mice had normal motor activity but displayed anxiety-like behavior and failed to recognize social novelty.

### The effects of *T. gondii* infection on olfactory sensitivity varied depending on sex

**Fig. 8:**
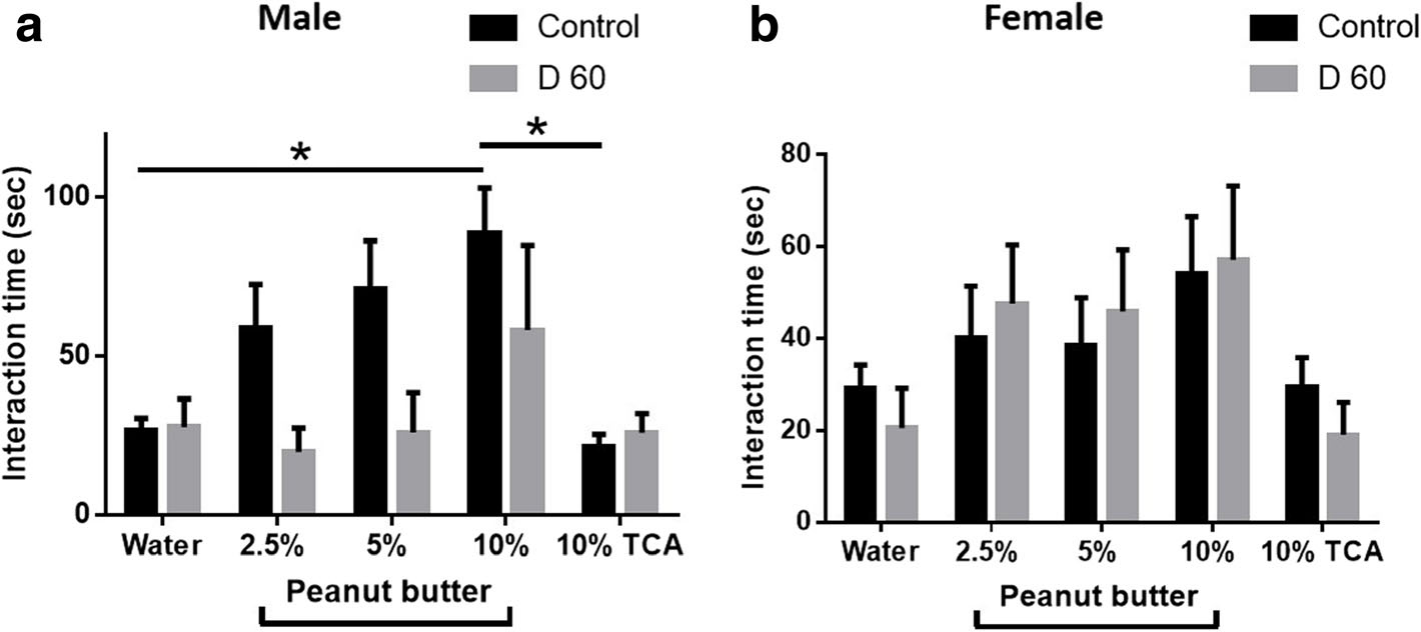
*T. gondii* infection reduced olfactory sensitivity exclusively in male mice. **a** Mice were tested once at day 60 post *T. gondii* infection. The time control and infected male mice spent sniffing each scent is shown. *n* = 6 (control group: 6 males; experimental group: 6 males). **b** The time control and infected female mice spent sniffing each scent is shown. *n* = 11–12 per group (control group: 12 females; experimental group: 11 females). Data are shown as mean ± SEM. *p < 0.05

Since VGLUT2 expression was reduced in *T. gondii-*infected mice and since olfactory dysfunction is one of the early indicators of AD, we examined the effects of *T. gondii* infection on olfactory sensitivity on the same group of animals in which social behavior was measured using a protocol that has been described previously [22]. The effect of *T. gondii* infection on olfactory sensitivity varied depending on sex. In contrast to infected male animals, mock-infected males showed a clear concentration-dependent preference for the peanut butter solution and a clear dislike for the aversive scent, trichloroacetic acid (TCA), indicating a loss of olfactory sensitivity in male mice infected with *T. gondii* ((*F*(1,75) = 6.800, *p* = 0.0110, two-way ANOVA, Fig. 8a). Overall, control female mice spent less time sniffing each scent compared to control male mice, although they did show a preference for the 10% peanut butter solution over both water and the aversive scent, TCA (Fig. 8b). Infected female mice displayed similar behavior compared to their respective controls and there was no significant main effect of experimental condition (*F*(1,105) = 0.0005111, *p* = 0.9820, two way-ANOVA, Fig. 8b), indicating that olfactory sensitivity in infected female mice was not significantly affected. Thus, olfactory sensitivity was reduced in male mice but it is intact in female mice.

We did not observe sex-specific differences in expression or protein levels of VGLUT1/2, NMDAR, or GAD67. We also did not observe sex-specific differences in any of the other behavioral measures we used, indicating that in our model of *T. gondii* infection sex-dependent differences are limited to olfactory sensitivity.

### *T. gondii* infection severely impaired spatial memory

**Fig. 9:**
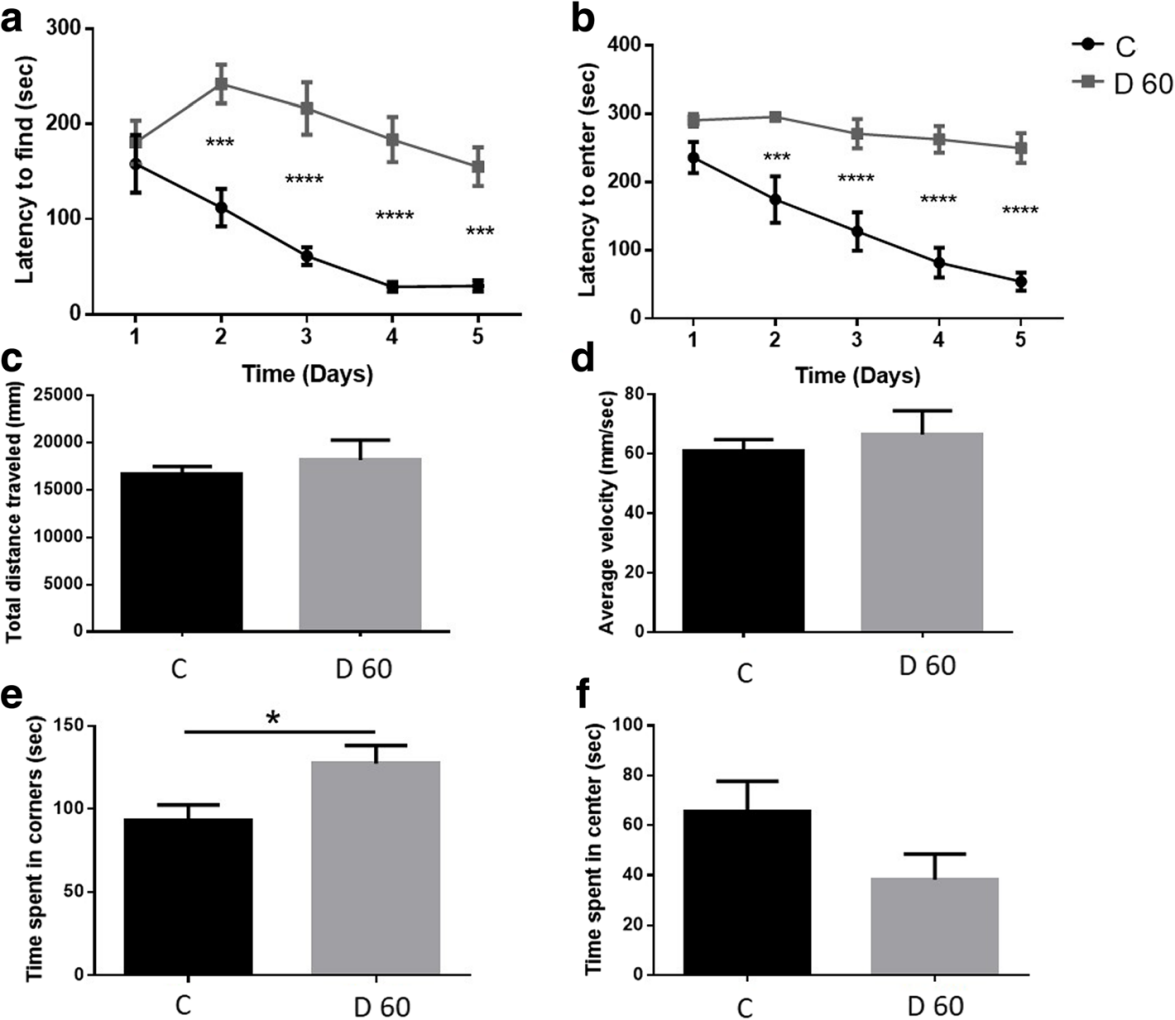
*T. gondii* infection severely impaired spatial memory but did not compromise motor coordination. Control and *T. gondii*-infected mice were tested in the Barnes maze for five consecutive days starting at day 60 post infection. The time taken by the mice to find the escape box (**a**) and the time taken to enter the escape box (**b**) are shown. *n* = 8–10 per group (control group: 4 males and 4 females; experimental group: 7 males and 3 females). **c** The total distance traveled, **d** the average velocity, and **e** the amount of time spent in the corners and in the center **f** of the open field during the 5-min observation period were recorded. Mice were tested once on the last day of Barnes maze testing. *n* = 8–10 per group (control group: 4 males and 4 females; experimental group: 7 males and 3 females). Data are shown as mean ± SEM. *p < 0.05, ***p < 0.001, **** *p* < 0.0001

The brains of *T. gondii*-infected mice showed a significant reduction in the expression of NMDAR, which is heavily involved in the formation of spatial and contextual memories. We tested the effect of *T. gondii* infection on spatial learning and exploration in the Barnes maze using a new cohort of mice. Control and infected animals were tested twice per day for five consecutive days. Testing sessions were separated by 15 min and data from both sessions were averaged for analysis. A main effect of time revealed that performance of the control mice improved over the 5-day testing period (*F*(4,64)  =  7.045, *p*  <  0.0001, two-way repeated measures ANOVA), while infected mice showed only a mild improvement in remembering the escape box location by day 5 of testing (Fig. 9a). Control mice were able to find and enter the escape box in significantly less time compared to infected animals at days 2, 3, 4, and 5 of testing (*F*(1,16) = 51.32, *p*  <  0.0001, two-way repeated measures ANOVA), indicating that spatial learning and memory in *T. gondii*-infected mice were severely impaired (Fig. 9a, b). These same animals moved a similar distance (*p* = 0.5222, two-tailed Student’s *t* test, Fig. 9c) and moved at a similar velocity (*p* = 0.5477, two-tailed Student’s *t* test, Fig. 9d) in the open field compared to the control mice, indicating that motor deficits cannot explain the impairment in spatial memory we observed. As with previous experiments, infected mice spent significantly more time in the corners of the open field (*p* = 0.0297, two-tailed Student’s *t* test, Fig. 9e) and less time in the center (*p* = 0.1028, two-tailed Student’s *t* test, Fig. 9f), indicating anxiety-like behavior.

## Discussion

The link between *T. gondii* infection and AD has not been fully elucidated, and epidemiological studies are contradictory. One study found that patients with AD had higher seropositivity rates for anti-*T. gondii* IgG antibodies compared to healthy controls, indicating a possible link between *T. gondii* infection and AD [54]. Two other studies reported no difference in the presence of anti-*T. gondii* IgG antibodies between AD patients and their healthy counterparts [55, 56]. Moreover, countries with high *T. gondii* seropositivity rates do not have higher rates of AD. For example, France had a *T. gondii* seroprevalence of 70% in the 1970s [57], but an AD prevalence of only 3.0% among French people ≥ 60 years of age in 2012 [58].

While *T. gondii* may not be the underlying cause of AD in the general population, it may initiate pathological events that over a life time can result in AD-like symptoms. We present direct evidence that *T. gondii* causes major signs of AD in mice that are not genetically manipulated towards AD (wild-type mice). We showed that infected wild-type mice showed major signs of AD including Aβ immunoreactivity, pTau expression, neuronal dysfunction, and behavior alterations. There is overwhelming evidence indicating that Aβ accumulation in the brain is the cause of neurodegeneration. In animal models, Aβ monomers and oligomers cause cognitive impairment, synaptic dysfunction, and neuronal loss [59]. In cultured neurons and astrocytes, administration of Aβ-impaired glutamate uptake [60] which could hinder neurotransmission, memory formation, and learning. Treatment of mouse cortical neurons with Aβ oligomers (trimmers or tetramers) induced neuronal toxicity and death [61]. We showed that Aβ immunoreactivity coincided with a decrease in NMDAR signal in the brains of infected mice as early as day 15 post infection. We conclude that *T. gondii* induced Aβ immunoreactivity which led to NMDAR loss. The loss of NMDAR disrupts the feedback inhibition signal by NMDA, GABA and VGLUTs and increases glutamate in the synaptic cleft, leading to neurotoxicity and neurodegeneration. Aβ enhances the endocytosis of NMDARs, thereby reducing their surface expression [19]. Alternatively, *T. gondii* infection led to reduced NMDAR expression which then led to Aβ immunoreactivity. It is also possible that the inflammatory response caused by *T. gondii* infection caused Aβ immunoreactivity and eventual neuronal loss. Acute infection with *T. gondii* attracts neutrophils, microglia, and dendritic cells to the site of the infection which leads to the release of cytokines necessary to promote killing of the parasite and inhibit its replication [62]. Inflammatory cytokines have also been implicated in the formation of Aβ plaques [63, 64] and neuronal cell death [65].

Our findings that *T. gondii* infection led to of Aβ immunoreactivity contradict two recent studies. The first one showed that *T. gondii* infection reduced Aβ plaque load in an AD transgenic mouse model (5xFAD) due to the recruitment of highly phagocytic monocytes [66]. The second one found that the ME49 strain of *T. gondii* reduced plaque deposition, neurodegeneration, and improved cognition in Tg2576 mice [67]. It is notable that both of these studies used AD transgenic mouse models. The observed reduction in plaque burden could be the result of *T. gondii*-driven clearance by hyperactivated astrocytes and microglial cells, as postulated by [66], or the result of *T. gondii*-driven acceleration of advanced signs of AD with resultant induction of cerebral amyloid angiopathy (CAA), which is the deposition of Aβ plaques in CNS vessels. However, neither study assessed the appearance of CAA or evaluated other advanced signs of AD. Alternatively, plaque formation could be a transient effect that Jung et al. missed by assessing plaque load and behavior in 9-month-old mice at 6 months post infection [67]. In our studies, Aβ immunoreactivity and behavior were assessed within 60 to 90 days post infection in wild-type mice.

In addition to molecular signs, AD is characterized by alterations in social communication and social memory [53]. We found clustering of *T. gondii* cysts and Aβ plaque formation in brain areas known to affect social behavior, namely the hippocampus and the prefrontal cortex [50, 51]. In line with these findings, we observed alterations in social novelty recognition in *T. gondii*-infected mice. This is consistent with other studies that have shown altered social behavior in mice genetically modified to overexpress Aβ [68]. Since the animals had normal activity levels in the open field, hyper- or hypoactivity or motor defects are unlikely contributors to the altered recognition of social novelty.

*T. gondii*-infected mice showed a severe impairment in spatial learning and memory as assessed by Barnes maze. These mice showed normal activity levels, indicating motor defects are not responsible for the spatial memory defect. *T. gondii* has been consistently associated with behavioral changes in mice [11, 69, 70]. In humans, some studies show effects of *T. gondii* on cognitive decline. For example, Gajewski et al. found that adults with *T. gondii*-positive antibody status performed poorly in a verbal memory test and showed reduced working memory [71]. However, the effects of *T. gondii* on cognition seem to depend largely on educational level, economic status, and ethnicity [10, 72].

We observed profound loss of VGLUT2 in olfactory sensory neurons indicating that there is also profound olfactory sensory neuronal loss which is one of the early signs of AD. The discrete upregulation of GAD67 in areas prevalent with *T. gondii* cysts, and increased VGLUT1 expression, which occurred near *T. gondii* cysts, may be a compensatory response to overcome neural inhibition and re-establish neural transmission. David et al. demonstrated that infection with *T. gondii* leads to a reduction in the primary astrocytic glutamate transporter GLT-1, increased extracellular concentrations of glutamate, and reduced dendritic spines [36]. They also reported no induction of neuronal cell death by the parasite as well as reduced expression of VGLUT1, which contradicts our results. These authors used 20 *T. gondii* cysts to infect mice, while we used only 10. It is possible that the higher MOI used by David et al. induced excessive stimulation of glutamatergic signaling resulting in excitotoxicity. In fact, they observed synaptic changes in the prefrontal cortex including reduced spine density at 6 weeks post infection, which has been shown to contribute to cognitive decline [36]. They also observed reduced brain wave activity as well as reduced expression of neuronal synaptic markers. We propose that Aβ induction and the high prevalence of cysts in the olfactory bulb contributed to loss of VGLUT2 on olfactory neurons, resulting in the upregulation of VGLUT1 and discrete increases in GAD67 to compensate for breakdown in neurotransmission at these sites. Together, these studies show that *T. gondii* infection caused loss of neurotransmission leading to severe neurodegeneration and major signs of AD in wild-type mice.

We also observed alterations in olfactory sensitivity which varied depending on sex. Mock-infected males spent more time inspecting all the tested scents compared to the female controls. This correlates with a previous study showing that male mice have a superior ability to discriminate between various odors compared to females [73]. Olfactory sensitivity in infected males was reduced compared to mock-infected animals but was unaltered in female mice. *T. gondii* causes a mild attraction towards cat urine in female *BALB/c* mice [74], a finding that would indicate intact olfactory sensitivity. A second study showed that only infected females but not males exhibit an attraction for cat urine [75], which correlates with the unaltered olfactory sensitivity we observed in our infected female mice and the reduced olfactory sensitivity we see in male animals.

**Fig. 10:**
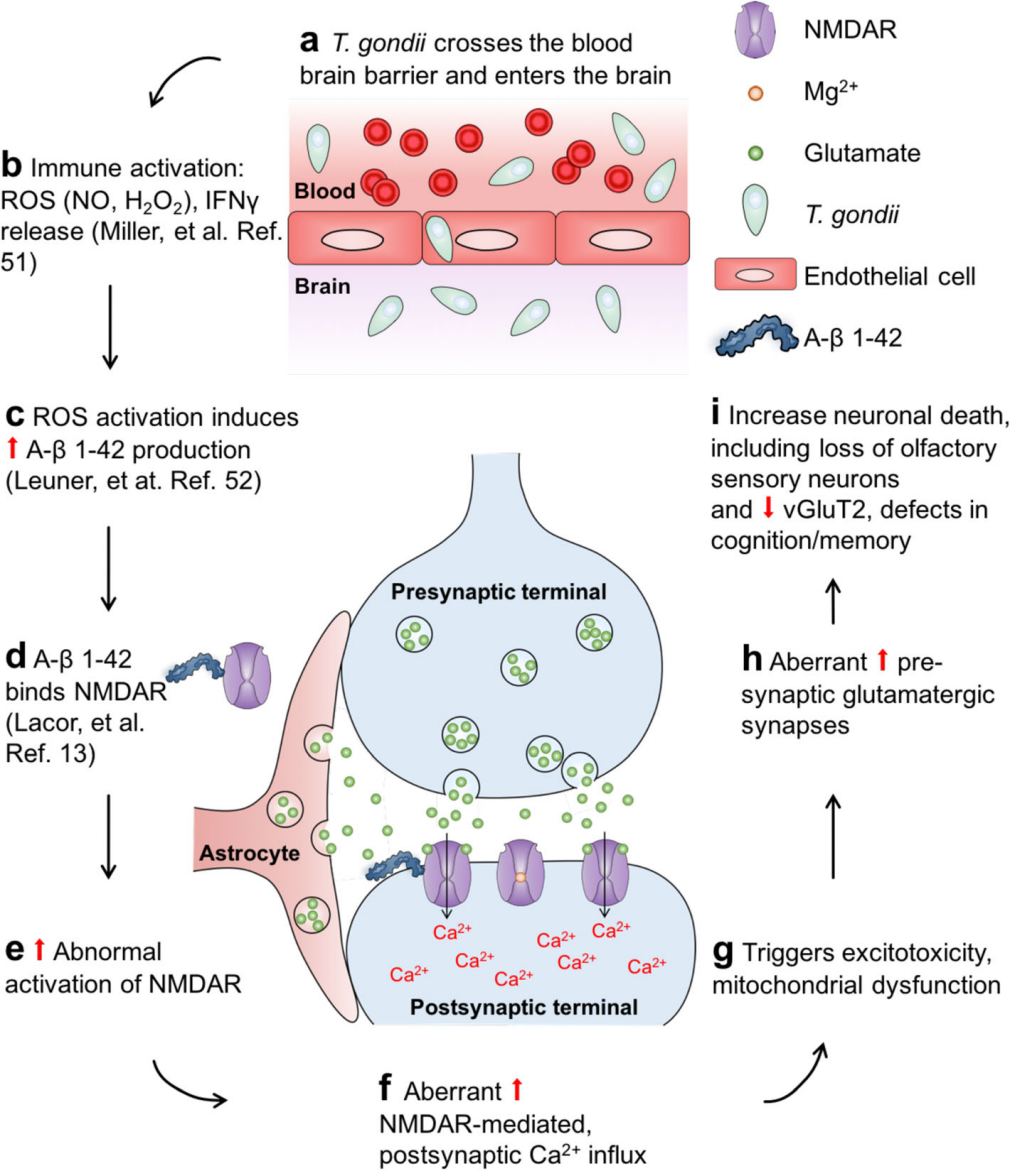
Working model describes how *T. gondii* possibly leads to early signs of AD. **a**, **b** *T. gondii* infection in the brain causes immune responses such as activation of reactive oxygen species (ROS) and release of interferon gamma (IFNγ). **c**–**e** Activated ROS induces A-β 1–42 production in the brain, which binds NMDAR and leads to abnormal NMDAR activation [14]. **f** Aberrant NMDAR activation results in a dramatic increase in Ca^2+^ influx in postsynaptic neurons. **g**–**i** This triggers toxic pathways that result in a pathological increase in presynaptic glutamate release, which then leads to loss of olfactory sensory neurons, reduction in VGLUT2, and, ultimately, early signs of AD

*T. gondii* infection in the brain activates reactive oxygen species (ROS) and causes the release of interferon gamma (IFNγ) [76]. We propose that ROS induces A-β 1–42 production [77], which binds NMDAR and leads to abnormal NMDAR activation [14] (Fig. 10). Aberrant NMDAR activation results in a dramatic increase in Ca^2+^ influx in postsynaptic neurons [78]. This triggers an excitotoxic insult that results in pathological increases in presynaptic glutamate release, resulting in neuronal damage, loss of olfactory sensory neurons, reduction in VGLUT2, and, ultimately, early signs of AD which include loss of olfactory sensitivity and memory impairments (Figs. 8 and 9).

To the best of our knowledge, we are the first to show that *T. gondii* directly induces AD signs in non-genetically manipulated animals. This study is likely to inform many areas of science including, infectious disease, neuroscience, neuroimmunology, immunology, and neurodegeneration and behavior.

## Additional files

Additional file 1: **Figure S1.** Specificity of the 6E10 and BAG-1 antibodies. Representative × 20 magnification images from control brains and brains from mice orally infected with 10 ME49 cysts for 60 days. Brains were collected after perfusion with ice-cold PBS. Control and infected brains were fixed and stained with a human anti-beta amyloid antibody, 6E10 (*green*) and an anti-*T. gondii* antibody BAG1 (*red*) and counterstained with DAPI (*blue*). A separate set of sections from *T. gondii*-infected brains were stained with isotype controls (mouse IgG and Rabbit IgG) to control for the specificity of the 6E10 and BAG-1 antibodies, respectively. A different set of sections from *T. gondii*-infected brains were stained for fluorescent secondary antibodies (anti-mouse 488 and Texas-Red anti-rabbit) to control for their specificity. Scale bar: 50 μm. (JPEG 114 kb)

Additional file 2: **Figure S2.** *T. gondii* induces Aβ immunoreactivity. A. Representative × 20 magnification images from control brains and brains from mice orally infected with 10 ME49 cysts for 30 (top) and 60 days (bottom). The inset shows a close-up image at × 63 magnification. Tissue sections were fixed and stained with an anti-*T. gondii* antibody BAG1 (*red*) and counterstained with DAPI (*blue*). A comparable brain section from the same animal was stained with a mouse anti-beta amyloid antibody (*green*) and counterstained with DAPI (*blue*). Scale bar: 50 μm. B. Representative × 20 magnification images from control brains and brains from mice orally infected with 10 ME49 cysts for 60 days. Tissue sections from the same animals used in A were fixed and stained with a human anti-beta amyloid antibody, 6E10 (*green*) and an anti-*T. gondii* antibody BAG1 (*red*) and counterstained with DAPI (*blue*). A separate set of sections from *T. gondii*-infected brains were stained with isotype controls (mouse IgG and Rabbit IgG) to control for the specificity of the 6E10 and BAG-1 antibodies, respectively. A different set of sections from *T. gondii*-infected brains were stained for fluorescent secondary antibodies (anti-mouse 488 and Texas-Red anti-rabbit) to control for their specificity. Scale bar: 50 μm. (JPEG 303 kb)
