# Supplementary figures and images for: Toxoplasma gondii alters NMDAR signaling and induces signs of Alzheimer’s disease in wild-type, C57BL/6 mice
Source: J Neuroinflammation. 2018 Feb 23;15:57. doi: 10.1186/s12974-018-1086-8 (PMC5824585; doi:10.1186/s12974-018-1086-8)

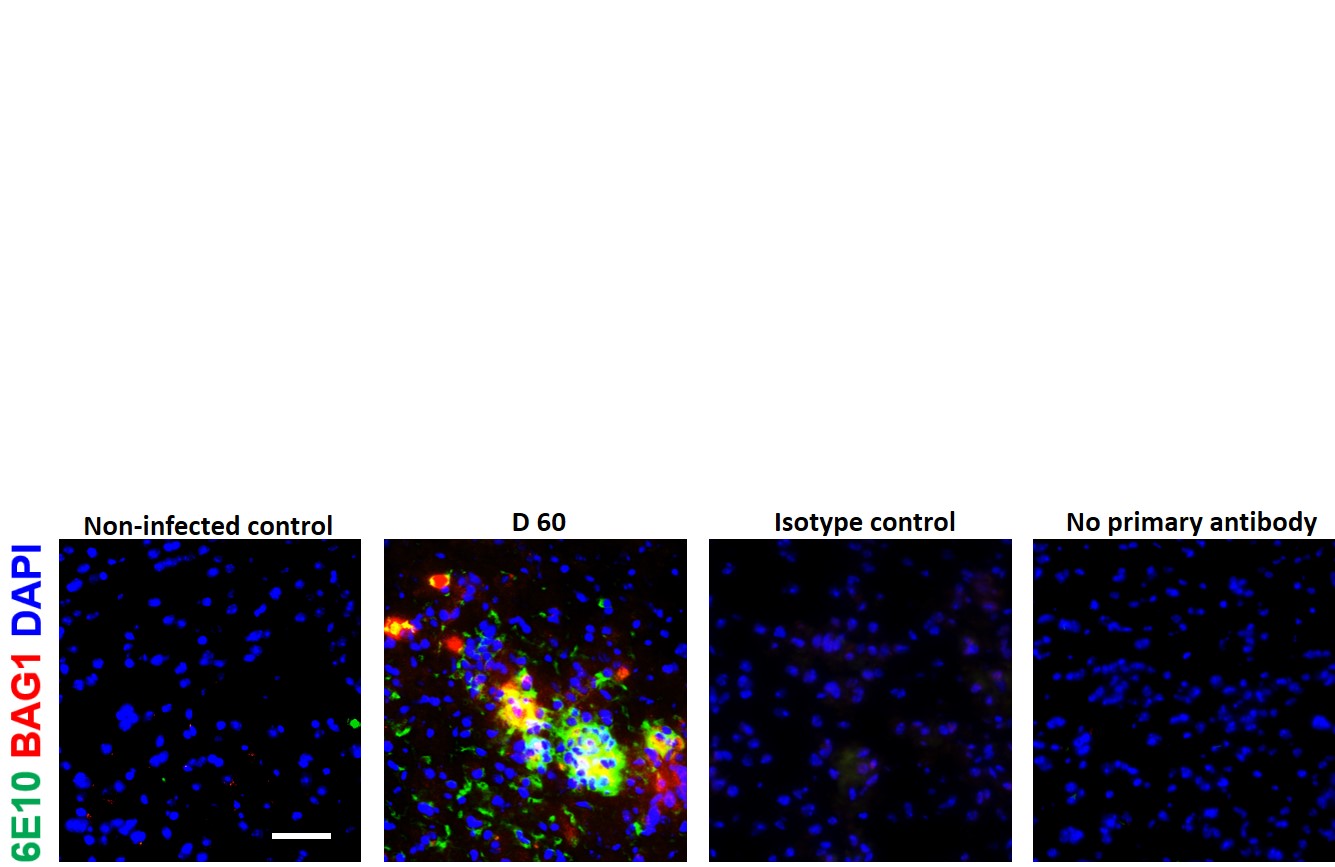

Supplement: Supplementary file 1 — Figure S1. Specificity of the 6E10 and BAG-1 antibodies. Representative × 20 magnification images from control brains and brains from mice orally infected with 10 ME49 cysts for 60 days. Brains were collected after perfusion with ice-cold PBS. Control and infected brains were fixed and stained with a human anti-beta amyloid antibody, 6E10 (green) and an anti-T. gondii antibody BAG1 (red) and counterstained with DAPI (blue). A separate set of sections from T. gondii-infected brains were stained with isotype controls (mouse IgG and Rabbit IgG) to control for the specificity of the 6E10 and BAG-1 antibodies, respectively. A different set of sections from T. gondii-infected brains were stained for fluorescent secondary antibodies (anti-mouse 488 and Texas-Red anti-rabbit) to control for their specificity. Scale bar: 50 μm. (JPEG 114 kb) [file 12974_2018_1086_MOESM1_ESM.jpg]

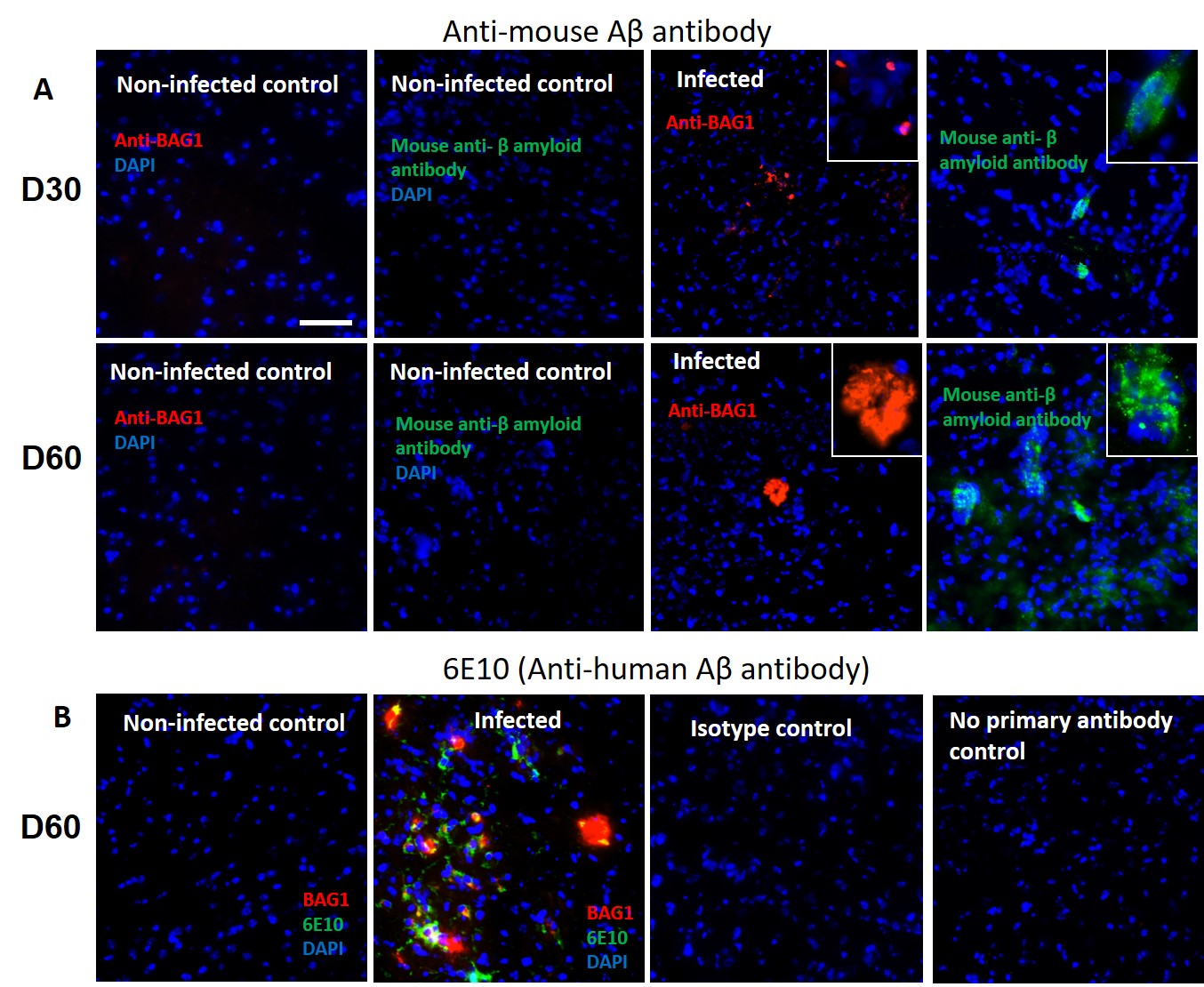

Supplement: Supplementary file 2 — Figure S2. T. gondii induces Aβ immunoreactivity. A. Representative × 20 magnification images from control brains and brains from mice orally infected with 10 ME49 cysts for 30 (top) and 60 days (bottom). The inset shows a close-up image at × 63 magnification. Tissue sections were fixed and stained with an anti-T. gondii antibody BAG1 (red) and counterstained with DAPI (blue). A comparable brain section from the same animal was stained with a mouse anti-beta amyloid antibody (green) and counterstained with DAPI (blue). Scale bar: 50 μm. B. Representative × 20 magnification images from control brains and brains from mice orally infected with 10 ME49 cysts for 60 days. Tissue sections from the same animals used in A were fixed and stained with a human anti-beta amyloid antibody, 6E10 (green) and an anti-T. gondii antibody BAG1 (red) and counterstained with DAPI (blue). A separate set of sections from T. gondii-infected brains were stained with isotype controls (mouse IgG and Rabbit IgG) to control for the specificity of the 6E10 and BAG-1 antibodies, respectively. A different set of sections from T. gondii-infected brains were stained for fluorescent secondary antibodies (anti-mouse 488 and Texas-Red anti-rabbit) to control for their specificity. Scale bar: 50 μm. (JPEG 303 kb) [file 12974_2018_1086_MOESM2_ESM.jpg]
